# Aggressive Behaviour of *Drosophila suzukii* in Relation to Environmental and Social Factors

**DOI:** 10.1038/s41598-020-64941-1

**Published:** 2020-05-12

**Authors:** Maria Belenioti, Nikolaos Chaniotakis

**Affiliations:** Laboratory of Analytical Chemistry, Department of Chemistry, University of Crete, Vassilika Vouton, Heraklion, 70013 Greece

**Keywords:** Ecology, Zoology

## Abstract

Aggression plays a crucial role in survival all across the animal kingdom. In this study, we investigate the aggressive behaviour of *Drosophila suzukii*, a known agricultural pest. Bioassays were performed between same sex pairs and the effect of environmental (food deprivation, sex, age and photophase) and social factors (non-social and social). Initially the inter-male and inter-female aggression was determined ethologically consisting of several behaviour patterns. Two hours starvation period increase locomotor activity of flies, promoting increased aggressive behaviour. Most of the behavioural patterns were common between males and females with a few sex-selective. Number of male encounters was higher in flies held in isolation than in those that had been reared with siblings whereas in case of females, only those that were isolated exhibited increased aggression. Females and males *D. suzukii* that were 4-day-old were more aggressive. In addition it is found that on the 3^rd^ hour after the beginning of photophase, regardless of age, both males and females rise to high intensity aggression patterns.

## Introduction

Aggression plays a crucial role across the animal kingdom. Its main role is to support the acquisition and the defense of limited resources such as food, mates and territories^1^. For males the use of aggressive behaviour in order to secure food resources influences greatly their daily life. Aggressive behaviour improves their overall health, their survival rate and allows them to pass their genetic material to their offspring^1,2^.

Aggressive behaviour in Drosophila species can be affected by environmental, social and genetic factors^1,3,4^. For this reason factors such as sex, age, fight experience and cuticular hydrocarbons have been studied in detail for a number of species^1,5–7^. In particular, cuticular hydrocarbon pheromones, such as 11-cis-vaccenyl acetate (cVA) and (Z)-7-tricosene promote aggression^8,9^. It has been shown that same sex pairs of Drosophila species exhibit aggression using gender-specific behaviour patterns^10,11^. As such, it is often the case that fights between *D. melanogaster* males begin with opponents raising both wings, then approaching each other and expressing offensive actions such as tapping, lunging, tussling and boxing^10^. Females on the other hand are in general less aggressive as they use mainly tapping and staring actions against each other^11^. Males with prior fighting experience are more successful in overpowering the opponent during an aggressive incident^7,12^. As a result winners are more likely to win again and losers are likely to lose^12^. Males that are 3-day-old are more aggressive when they compete with males that are 1-day-old^7^. Social isolation of fruit flies is shown to increase their aggressive behaviour and it is thus considered a significant passive stressor factor^7,13,14^.

Although aggressive behaviour has been reported for many different species^15^ it has not been described yet for the spotted wing Drosophila, *D. suzukii (Diptera: Drosophilidae)*^16^. This pest has spread widely in North America, Asia and very recently in Europe^17^. It is highly polyphagous and able to infest a wide range of both cultivated and wild fruits in different families such as cherry, berry, strawberry, apricot and grapes^18^. *D. suzukii* females have a serrated ovipositor which enables them to deposit fertilized eggs inside the ripening fruits. That infection induces a lesion which causes fruit to be unsuitable for sale and consumption^19^. During the 2010 crop period in northern Italy alone the economic loss from this pest was estimated to be over 500.000€, while in 2011 increased dramatically and was estimated at 3.000.000€^20^.

In this study the aggressive behaviour of *D. suzukii* were examined in detail. Various behavioural patterns similar to *D. melanogaster* and *D. simulans* were analysed. Environmental parameters, such as food deprivation, sex, age, photophase and social experience are examined and found that aggressive behaviour is affected by them.

## Results

### Effect of starvation

**Figure 1:**
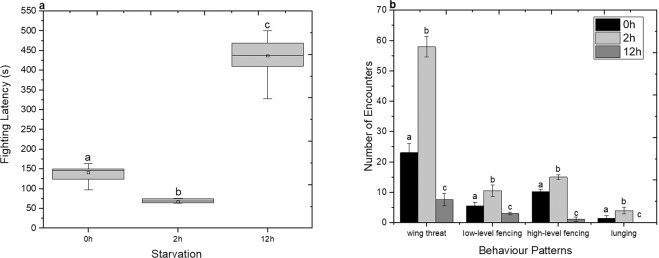
Effect of starvation on aggression of *D. suzukii* non-social males. (**a**) Aggression was quantified with the fighting latency. Grey columns indicate the median ± 25% area. The center line indicates the median value of data. The minimum and the maximum value represented by whiskers. Statistical difference was evaluated by One Way ANOVA Test and the significant level was at p < 0.05 (n = 25). Different letters indicate statistical differences. (**b**) Aggression was quantified with the frequency of behaviour, which is the number of encounters within 3600 s. Non-starvation in black bars, 2 h starvation in light grey bars and 12 h starvation in grey bars (mean ± s.d). Statistical difference was evaluated by One Way ANOVA Test and the significant level for both cases was at p < 0.05 (n = 25). Different letters indicates significant differences.

Food deprivation is often deemed to increase aggressive behaviour in various invertebrates and vertebrates^21–23^. When the food resources are sparse, individuals exhibit increased aggression in order to secure more food^24^. Previous published studies have shown that non-social Drosophila males are more aggressive compared to the non-social females^4,6^. Here the effect of food deprivation on aggression was examined under (a) non-starvation, (b) 2 h starvation and (c) 12 h starvation conditions. The aggression of non-social (single reared flies from the pupal stage) males *D. suzukii*, is quantified using the fighting latency within 3600 s (time taken to initiate fighting). As shown in Fig. 1a, the fighting latency decreased significantly (One-Way ANOVA, F(2, 72) = 12,261.6 p < 0.05) when flies were starved for 2 h. Interestingly, the effect of the long-term food deprivation (12 h) results in a significant increase of fighting latency compared to both 0 h and 2 h starvation phase. The latency for none and 2 h starvation was between 97 to 163 s and 62 to 76 s respectively, with a mean value of 141 ± 25 s (mean ± standard deviation, s.d) and 67 ± 19 s (mean ± s.d) respectively. On the other hand, the latency for 12 h starvation was between 328 to 499 s, with a mean value of 437 ± 87 s (mean ± s.d).

Subsequently the offensive actions of paired non-social males were studied as a function of food deprivation conditions by measuring the frequency of individual aggressive events^25^. Fig. 1b shows the frequency of encounters using wing threats, low or high level fences and lunges exhibited by non-social males that were either well fed or had been held in 2 h or 12 h starvation. Flies which had been held in 2 h of starvation exhibited more frequent aggressive events than those that had been held in 12 h starvation or those that were well fed (One-Way ANOVA wing threat: F(2, 72) = 2,891.17 p < 0.05; low-level fencing: F(2, 72) = 204.04 p < 0.05; high-level fencing: F(2, 72) = 116.16 p < 0.05; lunging: F(2, 72) = 230.99 p < 0.05). Specifically, long term food deprivation reduces the individual frequency of aggressive patterns. As such, this finding contradicts the initial assumption that the long term of food deprivation would lead to higher intensity of aggression.

**Figure 2:**
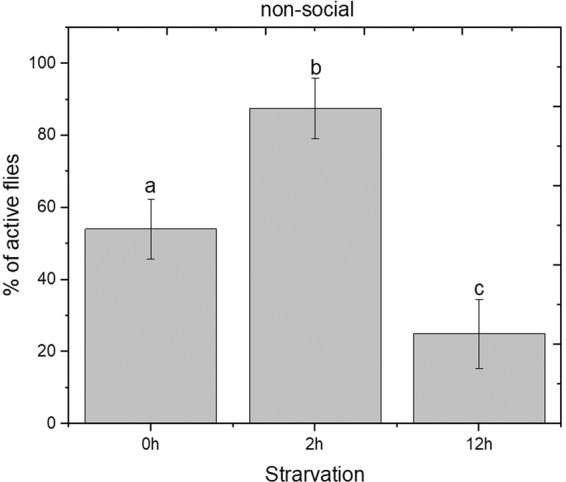
Effect of starvation in locomotion activity of *D. suzukii* non-social males. Activity was scored as the % of active flies within the 60 s. Statistical difference was evaluated One Way ANOVA Test and the significant level was at p < 0.05 (mean ± s.d) (n = 24). Different letters indicates significant differences.

Based on this observation, it was important to determine the effect of starvation on the locomotion. For this the locomotor effect of food deprivation in non-social males was evaluated using mobility assay^26^. A significant increase in locomotor activity between the 0 h and 2 h starvation phase was observed, followed by a significant decrease between the 2 h and 12 h starvation periods (One-Way ANOVA F(2, 9) = 50.7; p < 0.05) (Fig. 2). It is evident that the long-term starvation treatment reduces the locomotion which results in a reduction of aggressive behaviour. Changes on aggressive behaviour within starvation conditions were just as likely to be attributed to changes in locomotion.

### Ethogram of *Drosophila suzukii* aggressive behaviour

**Figure 3:**
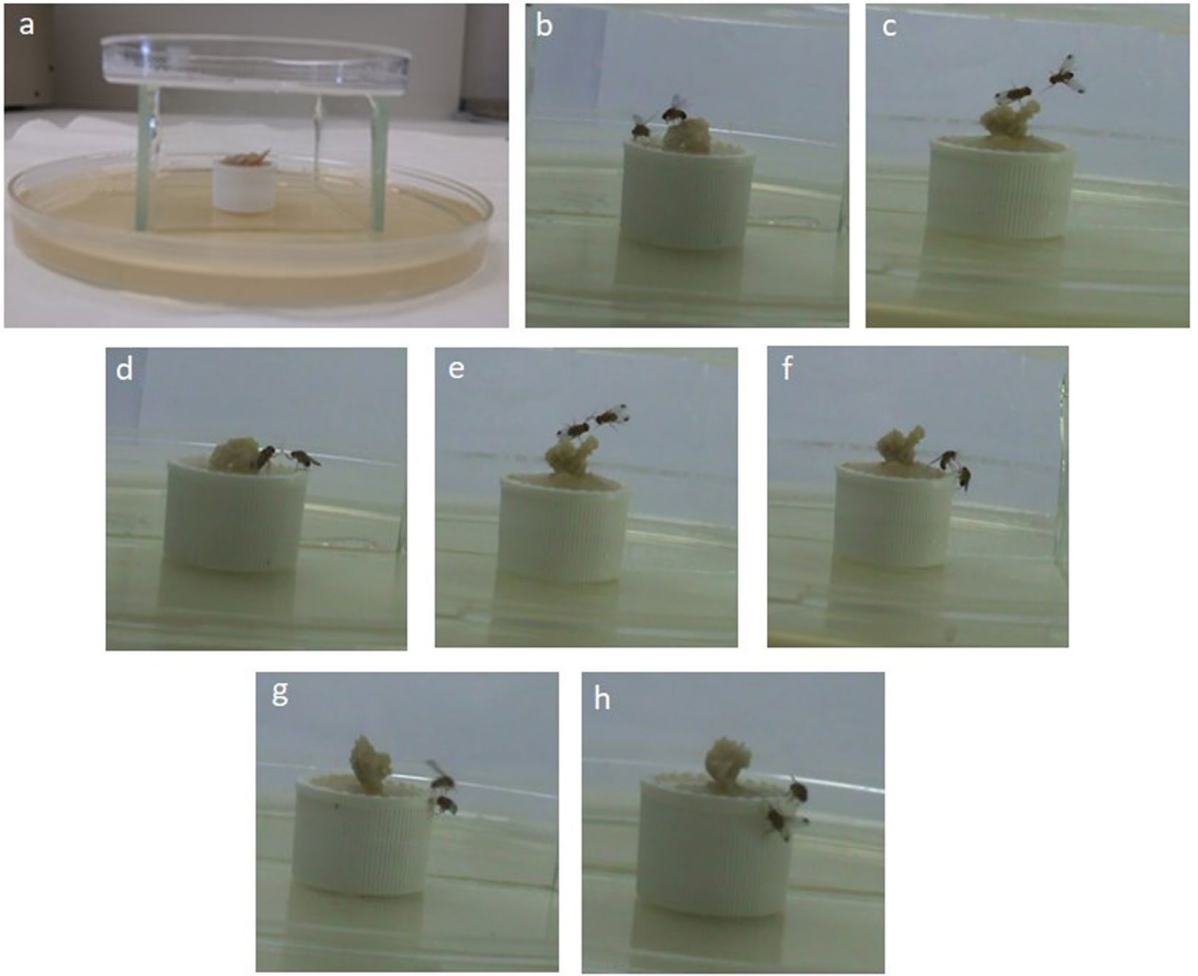
Photos taken by video analysis of non-social male *D. suzukii* aggression assay. (**a**) Experimental chamber. (**b**,**c**) Wing threat was observed from different optical angles. (**d**) Low-level fencing in which one fly extends his middle leg, and taps opponent’s leg. (**e**) High-level fencing in which both flies extend their forelegs simultaneously, (**f**) Lunging in which one fly rears up on its opposite legs and snaps down the opponent. (**g**) Flying attack in which male suddenly flies and attacks by thrusting its body over the opponent. (**h**) Defensive wing threat in which defender fly flicks its wing to a 45° angle.

Based on the aforementioned results, a 2 h starvation phase was applied to the rest of aggressive experiments. In an attempt to provide an entire determination of *D. suzukii* aggression, numerous fights between same sex pairs were performed and analyzed as shown in the ethogram of Table 1. In fact, the aggressive behaviour patterns were differentiated from the sex. A pair of females or males showed various types of aggressive behaviour in the observation chamber over the testing period (Fig. 3) (Supplementary, Video S1).

**Table 1:** Ethogram of offensive and defensive actions of male and female *D. suzukii* during aggressive encounters.

Male	Female
**Offensive Actions**	
Wing threat	
Both wings are raised simultaneously to a 45°–90° angle	Not observed
Tapping threat	
Fly extends the middle legs without touching the opponent	Fly extends the middle legs without touching the opponent
Approach	
One fly lowers his body and approaches (slow or fast) the opponent	One fly lowers his body and approaches (slow or fast) the opponent
Low - level fencing	
Fly extends one leg and taps opponent’s leg	Fly extend one leg and taps opponent’s leg
High – level fencing	
Both flies face each other, extend their leg and push the opponent	Both flies face each other, extend their leg and push the opponent
Feeding and Tapping	
Not observed	Fly extends his legs while feeding
Staring	
Not observed	Flies are staring each other
Chasing	
Fly runs after the other	Not observed
Lunging	
Fly rears up on his legs and snaps down the opponent	Not observed
Flying and attacking	
Fly flies and attacks by thrusting its body-weight over the opponent	Not observed
**Defensive Actions**	
Defensive wing threat	
Fly flicks both wings at 45° angle while is facing offensive action	Not observed
Walking away	
Fly retreats from food surface	Fly retreats from food surface
Flying away	
Fly flies away from food surface	Not observed

Offensive actions are separated in two categories: a) that in which the flies do not come in contact and b) that in which they come in contact with other individuals. When a pair of flies is introduced into the observation chamber, they express an initial aggression using specific actions but without any physical contact. These actions include the wing threat which involves the raising of the wings at a 45°–90° angle erection for <1 s, and a tapping threat in which the flies extend their middle legs without touching the opponent. Wing threats were also observed when flies approach each other during all types of offensive actions. Soon after flies tend to approach and chase each other (slowly or fast). During the approach one fly lowered his body and moves to the direction of the opponent and in the chasing, one fly runs after the other.

One of the most frequent offensive actions within the contact category is the movement of leg extensions, which can be either low or high level fencing. In the case when one fly extends the middle leg there is a low level fencing while when both flies extended their forelegs simultaneously there is a high level fencing action. Another observed offensive action was lunging, in which a male reared up the front legs and snapped down the other fly. Moreover a flying attack was observed in which male that is expected to win suddenly flies and attacks by thrusting its body over the opponent. At the same time the aggression intensity of the males could initiate at a low level, and escalate during the male-male interaction, although in some cases a high level aggression can be observed from the initial stages. Usually offensive actions are followed by grooming behaviour. During the encounters it is observed that weak fly (looser) falls backwards from the food cup onto the agar surface. The observed defensive actions include walking, running or flying away either from the opponent or from the surface of the food cup, accompanied usually with a flicking wing action.

Most of the behaviour patterns were common between males and females but some of them were sex-specific. Wing threat, chasing, lunging, flying attack, fall and flying away are typical of male insects. Both sexes displayed low or high level fencing but the duration of such behaviour was not always the same. Such actions usually lasted for more than 3 s in males, but were always less than 3 s in females. Low level fencing while feeding and staring at each other showed to be specific to females. Most often females extended one leg and tapped the opponent (low-level fencing), and on rare occasions they face each other (high-level fencing). Females were not as aggressive as were males toward each other.

### Environmental and social factors eliciting aggressive behaviour

**Figure 4:**
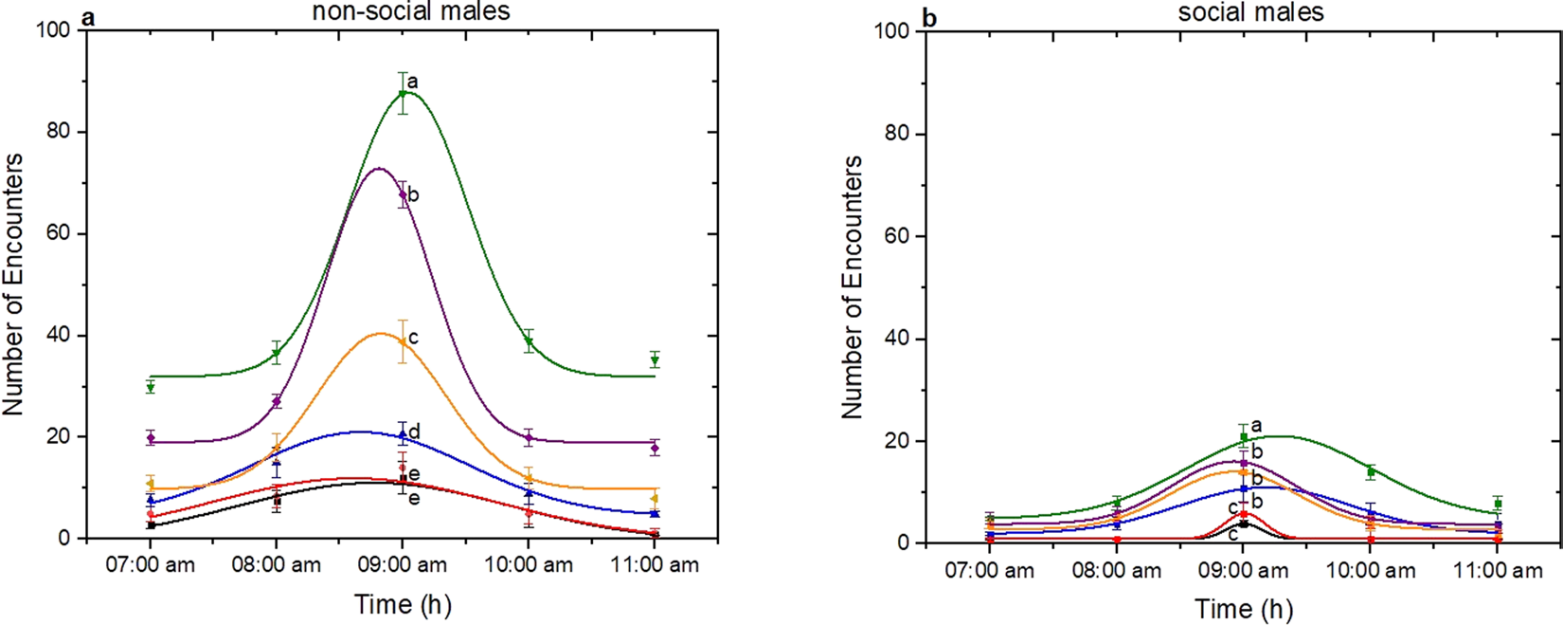
Effect of photophase and age on aggression of *D. suzukii* non-social and social males. Aggression was quantified as the sum of encounters’ number within 3600 s. For both social status six different fly ages (1d-black, 2d-red, 3d-blue, 4d-green, 5d-purpple and 6d-orange) were tested during five hours after the beginning of photophase (07:00 am to 11:00 am). Data were analyzed using Gaussian distribution and for a) non-social males 1d-R^2^ = 0.985, 2d-R^2^ = 0.923, 3d-R^2^ = 0.987, 4d-R^2^ = 0.962, 5d-R^2^ = 0.997, 6d-R^2^ = 0.971, and for b) social males 1d-R^2^ = 1, 2d-R^2^ = 1, 3d-R^2^ = 0.94564, 4d-R^2^ = 0.96358, 5d-R^2^ = 0.94307, 6d-R^2^ = 0.99298. Statistical difference was evaluated by One Way ANOVA Test and the significant level was at p < 0.05 (mean ± s.d) (n = 25). Different letters indicates significant differences.

The aggressive behaviour of many Drosophila species has shown to be affected both by social experience and environmental factors^1^. For this, aggression bioassays between same sex of *D. suzukii* flies are performed in order to establish how socialization influences the number and the kind of encounters. Bioassays were set up in such way as to study simultaneously the effect of age and the photophase. Six different fly ages (1d, 2d, 3d, 4d, 5d and 6d day old) were evaluated during five hours after the beginning of photophase (lights on at 07:00 am) of 12 h:12 h L:D (Light:Dark). Males that were up to 3-day-old regardless of social status and experiment control time exhibited low number of encounters compared to older males. Males that were 4-day-old performed the highest number of encounters (Fig. 4a for non-social males and 4b for social males). A decrease in the number of encounters was observed at the age of 5 and 6 days old. It was observed for example that at 09:00 am when males were 4-day-old of either social status, the number of encounters were higher than those exhibited by younger (1 and 2 days old) and older males (5 and 6 days old) (One-Way ANOVA, non-social: F(5, 114) = 13031.46, p < 0.05; social: F(5, 114) = 1154.01, p < 0.05). In addition, aggressive behaviour for all ages and for both social and non-social *D. suzukii* males varied during the course of the day. The time of experiment seems to have also a strong effect in aggression. During the first 2 h after the beginning of photophase (07:00 am and 08:00 am), the number of encounters of either age were low. After those hours the number of encounters reached a maximum value on 3^rd^ hour (09:00 to 10:00 am), followed by a sharp decrease (One-Way ANOVA, non-social: F(4, 122) = 13960.82; p < 0.05, social: F(4, 122) = 1816.39, p < 0.05).

**Figure 5:**
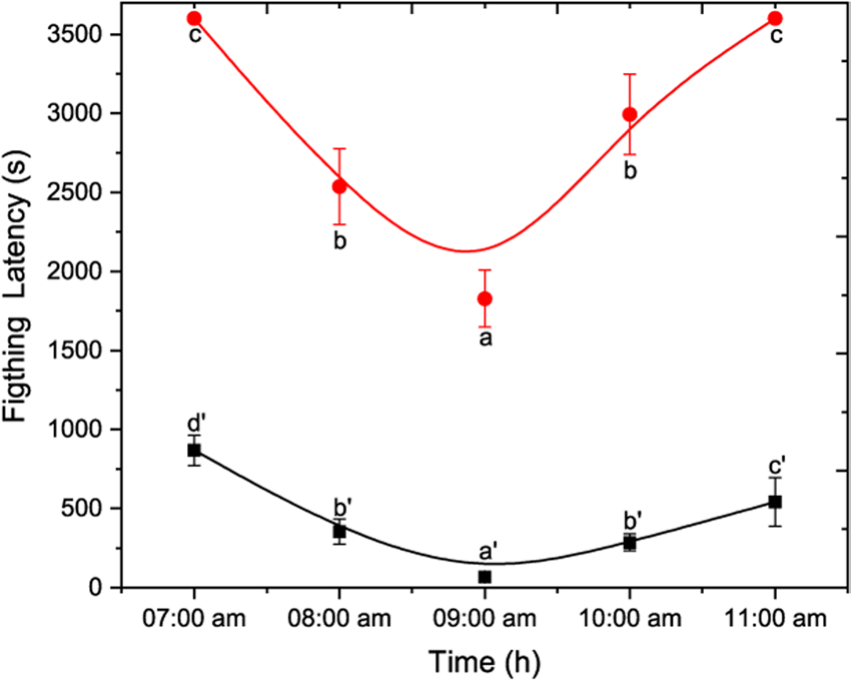
Effect of photophase on fighting latency of *D. suzukii* non-social and social males. Non-social in red line and social in black one. Statistical difference was evaluated by One Way ANOVA Test and the significant level was at p < 0.05 (mean ± s.d) (n = 25). Different letters indicates significant differences.

The effect of photophase in aggressive behaviour was confirmed with the quantification of fighting latency for both social and non-social 4-day-old males. It is found that fighting latency was lower on 3^rd^ hour after the beginning of photoperiod (09:00 am to 10:00 am) and was significantly different during the other times. This tendency was similar to both social and non-social males, as shown in Fig. 5 (One-Way ANOVA non-social males: F(4, 122) = 835.05; p < 0.05 social males: F(4, 122) = 419.43; p < 0.05).

Results of behaviour pattern’s analysis between either of social status are presented as supplementary material, Fig. S1. It was observed a significant increase in offensive actions (expressed as number of encounters) for non-social males compared with social ones (independent samples T-test, wing: t(48) = 140.01 p < 0.05; low-level fencing t(48) = 58.76 p < 0.05; high-level fencing t(48) = 99.45 p < 0.05 and lunging t(48) = 30.75 p < 0.05). In fact, the two high intensity offensive actions such as high level fencing and lunging were performed only in non-social males.

In Drosophila species, male aggression has been characterized in detail but little is known about female aggression^24^. As such, factors of age and photoperiod were also studied in *D. suzukii* females. Our data indicates that social female flies do not exhibit offensive actions. Subsequent experiments, therefore, were only used using non-social female flies.

**Figure 6:**
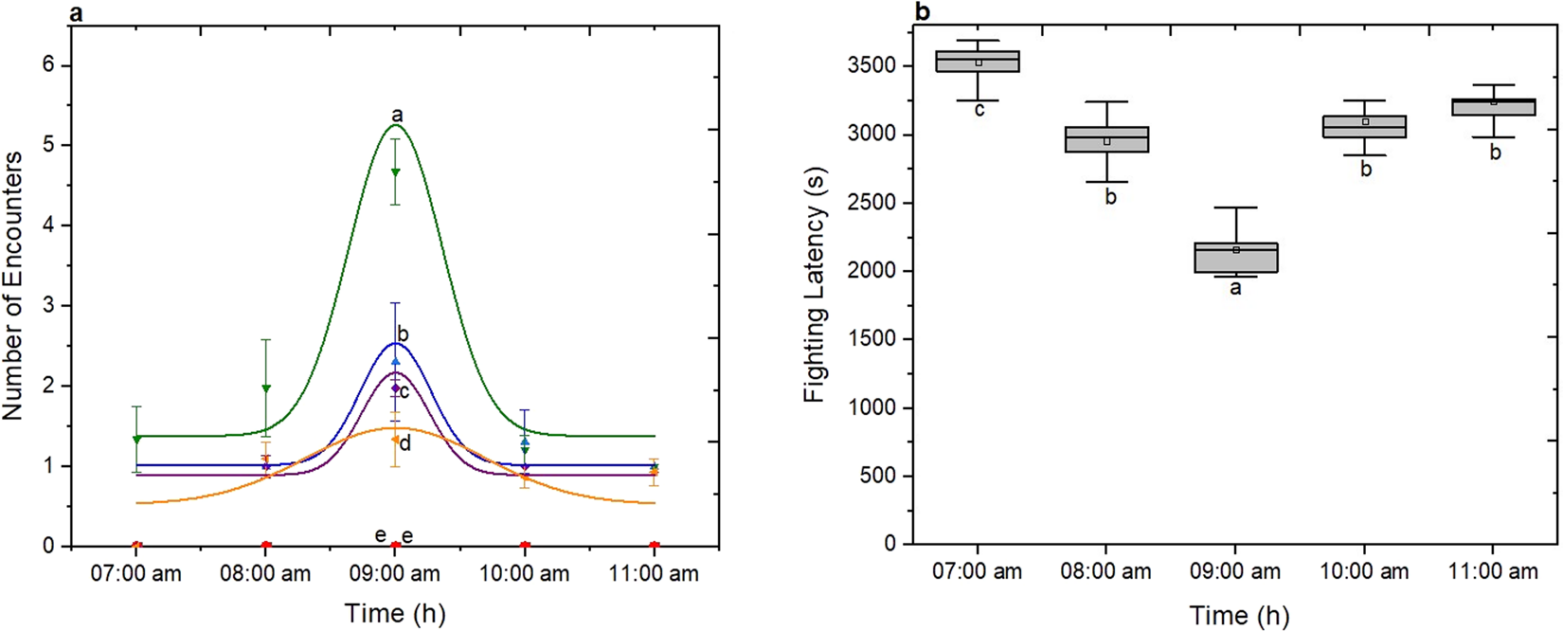
Effect of environmental factors on aggression of *D. suzukii* non-social females. (**a**) Aggression was quantified as the sum of encounters’ number within 3600 s. For both social status six different fly ages (1day-black, 2day-red, 3day-blue, 4day-green, 5day-purpple and 6day-orange) were tested during five hours after the beginning of photophase (07:00 am to 11:00 am). Data were analyzed using Gaussian distribution and for 3d-R^2^ = 0.945, 4d-R^2^ = 0.991, 5d-R2 = 0.897, 6d R^2^ = 0.932. The statistical difference was evaluated by One Way ANOVA Test and the significant level was at p < 0.05 (mean ± s.d) (n = 25). (**b**) Effect of photoperiod on fighting latency of *D. suzukii* non-social females. Grey columns indicate the median ± 25% area. The center line indicates the median value of data. The minimum and the maximum value represented by whiskers. Statistical difference was evaluated by One Way ANOVA Test and the significant level was at p < 0.05 (n = 25). Different letters indicates significant differences.

Age influenced both isolated females and males in similar way (One-Way ANOVA, non-social: F(5, 114) = 340.22; p < 0.05). Analysis of photophase’s factor indicates that non-social females showed the most aggressive behaviour on 3^rd^ hour after the beginning of the photophase (09:00 am) as shown in Fig. 6a (One-Way ANOVA, F(4, 122) = 47.21; p < 0.05). This is also the case when the fighting latency trend of the 4-day-old non-social females was studied as shown in Fig. 6b. The earliest expression of aggression for non-social *D. suzukii* females was observed 3 h after the beginning of photophase at 2,160 ± 185 s (mean ± s.d). The fighting latency on five different hours of photophase was significantly different (One-Way ANOVA F(4, 122) = 258.75; p < 0.05).

Finally, the fighting frequency is measured for both sex and both social status of *D. suzukii* as the percentage of pairs that show aggressive pattern during the observation period^27^. For non-social and social *D. suzukii* males, aggressive patterns were observed for 82 ± 2% and 49 ± 4% of pairs respectively, while for the non-social females this decreased to 23 ± 4% (mean ± s.d).

### Locomotion activity

**Figure 7:**
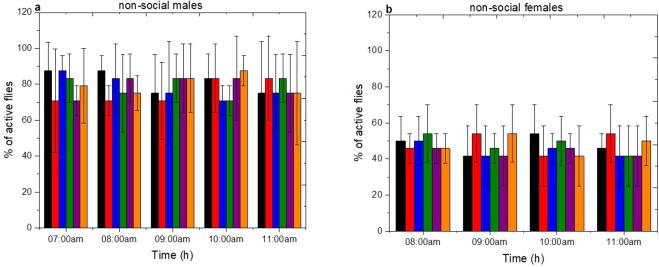
Locomotion activity of *D. suzukii* non-social males and females. Activity was scored as the % of active flies within the 60 s (mean ± s.d) (n = 24). The locomotion was tested for (**a**) non-social males and (**b**) non-social females in 6 different ages (1d-black, 2d-red, 3d-blue, 4d-green, 5d-purpple and 6d-orange) during five hours after the beginning of photophase (07:00 am to 11:00 am). There is little statistical difference p > 0.05.

In order to relate the effect of environmental and social factors to the spontaneous locomotor activity of *D. suzukii* a mobility assay method was employed^26^. The activity of *D. suzukii* at six different ages (1d, 2d, 3d, 4d, 5d and 6d day old) in relation to sex, age and social status was measured during five different hours after the beginning of photophase (photoperiod;12 h:12 h L:D) from 07:00 am to 11:00 am. The activity was scored as the % of active flies within the 60 s observation period. As it is shown in Fig. 7(a,b) non-social males and females displayed the same level of locomotion activity independent of age and photophase (One-Way ANOVA non-social males: F(29, 90) = 0.55 p = 0.97; non-social females: F(29, 90) = 0.52 p = 0.98). The same trend is observed in the case of the social males and females *D. suzukii* as shown in supplementary material (Fig. S2a,b).

## Discussion

While the aggression behaviour of Drosophila species has been extensively studied^1^ there are limited data for *D. suzukii* pest. The present *s*tudy examines the effect of social and environmental factors on aggressive behaviour of *D. suzukii*. Long term food deprivation was assumed to influence the aggression of flies in resource competition, but decreased the tendency to perform offensive actions, compared to short term effect of food (2 h) which increased the aggressive behaviour. At the same time the reduction in total activity observed after 12 h starvation period confirmed that reduced aggression on 12 h starved non-social males was due to the reduction of locomotion. This is probably due to the loss of strength and maybe dehydration, since starvation was performed in empty tubes. These results are in accordance with studies obtained in *D. melanogaster*, where a 24 h food deprivation period resulted in a decrease of the aggressive behaviour^24^. Several publications have been focused on the octopamine (OA) and tyramine (TA) function, in relation to this behaviour. It is a common understanding that without OA Drosophila males cannot go to higher levels of aggression^1^. In Drosophila larvae OA enhances locomotion during fight reactions or state of hunger, whereas TA reduces locomotion during the digest^28–32^. Nonetheless, the intense hunger might increase strive for food resources, but at the same time it may also increase the cost of aggression to defend food^33^. In the Economic Defensibility Theory^33^, resources are defended only if the benefit outweighs the cost, thus offensive behaviour will be suppressed if the perceived cost offsets the value of food acquisition. As a result, a 2 h starvation phase is long enough to make flies more active and so more aggressive without reducing their locomotion and their fighting ability. Based on the aforementioned results, the aggressive behaviour and the effect of the above factors were examined, following a 2 h starvation phase.

Comparing the ethogram of *D. suzukii* shown in Table 1 with that of the *D. melanogaster*^1^ there are many similarities and differences related to the aggressive behaviour of these two species. Both show the wings erect, which a threat display used by males and it is quite common also in *D. simulans*^10,16,34^. Approaching, chasing, fencing, lunging, wings vibration, flying and walking away^6,34^ are also common offensive patterns between these species. However, there are certain patterns displayed by *D. melanogaster* and *D. simulans* males which are not observed in the case of *D. suzukii*. These patterns includes the holding; where one fly holds the opponent and tries to immobilize it; boxing, where both flies raise up on their hind legs and hit each other with their forelegs and tussling; where both flies tumble over each other^10^. On the other hand, there is an offensive action of flying attack, which is displayed by *D. suzukii* males and is not observed in the case of *D. melanogaster* and *D. simulans*^34^. Moreover, it has been reported that *D. melanogaster* and *D. simulans* groom themselves after tussling and holding actions^34^, as it has also been observed in the case of *D. suzukii*.

Some of the observed *D. suzukii* behaviour patterns were the same between sex and some were male specific. Wing threat, chasing, lunging, flying attack, fall and flying away are not observed on female insects. The most common action of *D. suzukii* females upon the approach of another female was staring at each other and pushing off with their legs (fencing). Both of those patterns are also observed in *D. melanogaster* females^6^. Despite this, *D. suzukii* females are overall much less aggressive than their male counterparts. A similar trend has also being observed for the *D. melanogaster*^6,11^. This difference in the aggression between males and females has been attributed to the fact that males are more territorial than females in an effort to protect food resources as they do not usually share food with other individuals^6^.

The results of the experiments evaluating the influence of social interaction of *D. suzukii* on the aggressive behaviour of males showed that non-social males exhibit much higher aggression compared to social ones. These findings corroborate to the report suggesting that the social males become habituated and they are not influenced by the sensory signals produced by sibling males^35^. On the other hand only non-socialized females show aggressive behaviour in the observation chamber. However, it is interesting to note that females who are kept in a high density rearing vial showed the behaviour pattern of tapping. Published data have shown that Drosophila in rearing vials containing yeast, such as in ours experimental setup here, prior to the aggressive assay showed a less frequent aggressive behaviour compared to those close to food source without yeast^6^. The low aggression of social females can thus be attributed either to the actual composition of the food resource available, or to the low density of the individuals.

The age of flies seems to play a significant role in the behaviour of individuals. Both sex of *D. suzukii* at the early age (1d to 2d-old) did not show any significant aggression, this changed with ageing, exhibiting the highest number of aggressive encounters at the age of 4 days. Also, the results from locomotion assay showed that the locomotor of *D. suzukii* was the same independent of the age. As such, a possible explanation is the fact that at the age of four *D. suzukii* is reached sexual maturity. This is in agreement with published work showing that the highest amount of cuticular hydrocarbons (CHCs) is detected at that age^36^. As CHCs can influence the physiology and behaviour of interacting individuals within a group, they seem to play a significant role in the social behaviour of the flies^37^. A similar social behaviour has also been observed in the case of *D. melanogaster* males^7^.

Circadian rhythm is a controlling parameter in the pheromone production of flies^38,39^. It has been observed that in case of Drosophila different kinds of behaviour, such as aggression^14^, locomotor activity^40^ mating^41^, and feeding^42,43^ is mediated by pheromonal signaling, and that intensity of such activities is related to the specific times of the day^44^. For example diurnal mating activity of virgin *D. suzukii* occurs three hours after onset of the photoperiod^37^. Simultaneously, *D. suzukii* showed a stable level of locomotion during the five hours after lights on. Thus, the circadian rhythm in correlation with the pheromone production could be the reason of the aggressive behaviour is at its peak during the third hour of photophase.

Understanding of *D. suzukii’s* intraspecific interactions may enhance to unveil the ecological niche of this pest. This knowledge could give new routes for Integrated Pest Management (IPM), since ecology of pest is the principle of it^45^. The challenge for the future is to study how cuticular hydrocarbons influence the aggression of *D. suzukii*, since pheromone profile is a major parameter in determining the insect behaviour^46^.

## Methods

### Rearing conditions of flies

The fruit fly *D. suzukii* was used throughout this study. Flies were maintained in the quarantine facility at the Laboratory of Entomology, Department of Biology, and University of Crete, Greece. Population was reared under an 12 h:12 h L:D photoperiod at 21 ± 2 °C temperature and 65 ± 5% relative humidity^47^. Insects were kept in sterile vials which contain a cornmeal diet that consisted of agar (5 g), sugar (11.6 g), fresh yeast (28.33 g) and cornmeal (33.33 g) in nanopure water (1 L), and heated to 60 °C for 5 hours under stirring. Nipagin (0.83 g) dissolved in ethanol (8.33 ml) were subsequently added to sterilize the diet prior to its distribution into the vials. To collect and maintain non-social flies, pupae were isolated in individual Eppendorf tubes containing 1 ml of cornmeal diet. For social flies, emerging flies were collected 1 h after eclosion and were kept grouped with same age and sex siblings in fresh food. The population density of social flies was about 20-25 insects per vial^7^.

### Aggression arena set-up

The experimental set up was previously described^3,48^ and aggression assays were performed in a square chamber placed on a Petri dish (9 cm internal diameter) containing a layer of agar (2 ml) solution (5%w/v) and a vial cup containing cornmeal diet and a drop of yeast paste in the center of the food surface. Agar solution and drop of dry yeast were used for extra humidity and attraction respectively^49^. A lid from a Petri dish (5 cm internal diameter) with a hole for insertion of flies was placed on the top of square chamber.

### Aggression assay

Aggression assays were performed between virgin same age and same sex pairs of males or females. Two randomly selected flies were removed either from isolation (Eppendorf tubes) or grouping vials (depend on examined factor) by aspiration and placed in the chamber arena through the hole in the top of lid, then it was covered in order to prevent their escape. All interactions between the two flies were scored and video recorded during over a period of 3600 s. The video recordings were analyzed visually in order to define the aggression. Aggression is quantified with the determination of fighting latency and the occurrence frequency of each behaviour pattern. If insects were paused the interaction between them for more than 2 sec and/or separated by more than two body lengths it would consider the end of encounter^11^. Latency of fighting is defined as the time until first fight occurs^24^. Frequency of behaviour patterns occurrence defined as the encounters number of each behaviour pattern. For every treatment, 25 independent randomly selected couples were tested. All bioassay experiments were carried out under well-controlled environmental conditions (temperature 21 ± 2 °C and relative humidity 65 ± 5%).

### Starvation treatment

Based on the already published results, non-social males were more aggressive as compared with non-social females^4,6^. Thus, the effect of food deprivation was tested only in non-social males (4-day-old) of *D. suzukii*. Effect of food deprivation was examined under a) non-starvation, b) 2 h starvation and c) 12 h starvation conditions. Two and twelve hours before aggression assay, non-social males were randomly selected for the deprivation treatment. They were moved by aspiration into an empty Eppendorf tube. Then pair of well fed, 2 h and 12 h flies was introduced into the observation arena and aggressive assay was performed at 09:00 am every day. Twenty five replicates per condition were tested (n = 25).

### Effect of environmental and social factors

Based on food deprivation’s results, study of aggression was took place following a 2 h starvation phase. Both sex of non-social and social *D. suzukii* were examined with aim to study the effect of socialization in insect. Simultaneously, six different ages (1d, 2d, 3d, 4d, 5d and 6 day old) during five different hours after beginning of photophase (07:00 am to 11:00 am) were examined with aim to study the effect of age and photophase in aggression of *D. suzukii*.

### Locomotion assay

Spontaneous locomotor activity of flies was measured using mobility assay^26^. A single fly was transferred by aspiration without anesthesia into an empty Petri dish (internal diameter 5 cm). Petri dishes were set out on a table, which is isolated by rubber base from vibrations eventually transmitted by the floor. Flies were kept for 900 s to be acclimatized and then each fly was observed as active or inactive. Activity was defined as any motion of the fly (walking and attempt to fly); grooming was considered as inactivity. Activity is quoted 1 and inactivity 0. Activity of flies was calculated as the percentage of active flies for each round of observation (six Petri dishes per round of observation). Duration of an observation period was 60 s. Temperature and humidity of the experimental room was 21 ± 2 °C and 65 ± 5% respectively. Twenty five experiments for starvation and social factor, sex, age and time of photoperiod were performed (n = 24).

### Statistical analysis

For the statistical analysis of the bioassays results, the reported mean and standard deviation (s.d) were calculated using software IBM SPSS Statistics 24. Raw data of aggression (n = 25) and locomotion experiments (n = 24) were analyzed with the Normality Test of Shapiro-Wilk (n ≤ 50). Then depending on the distribution type and the variables independent samples T-test and One Way ANOVA test were used. The p value was set at 0.05 corresponding to 95% confidence limit. Gaussian distribution was used to determine the effect of fighting latency.

## Supplementary information

Supplementary Information.

Supplementary Information 2.
