# Supplementary material for: Aggressive Behaviour of Drosophila suzukii in Relation to Environmental and Social Factors
Source: Sci Rep. 2020 May 12;10:7898. doi: 10.1038/s41598-020-64941-1 (PMC7217943; doi:10.1038/s41598-020-64941-1)
Supplement: Supplementary file 1 — Supplementary Information. [file 41598_2020_64941_MOESM1_ESM.pdf]

Aggressive Behaviour of *Drosophila suzukii* in Relation to Environmental and Social Factors

Maria Belenioti[1, +] **and Nikolaos Chaniotakis**[1,*, +]

[1]Laboratory of Analytical Chemistry, Department of Chemistry, University of Crete, Vassilika Vouton, Heraklion, 70013, Greece
[*]corresponding author chaniotakis@uoc.gr
[+]these authors contributed equally to this work

Supplementary materials

Supplementary Fig. S1

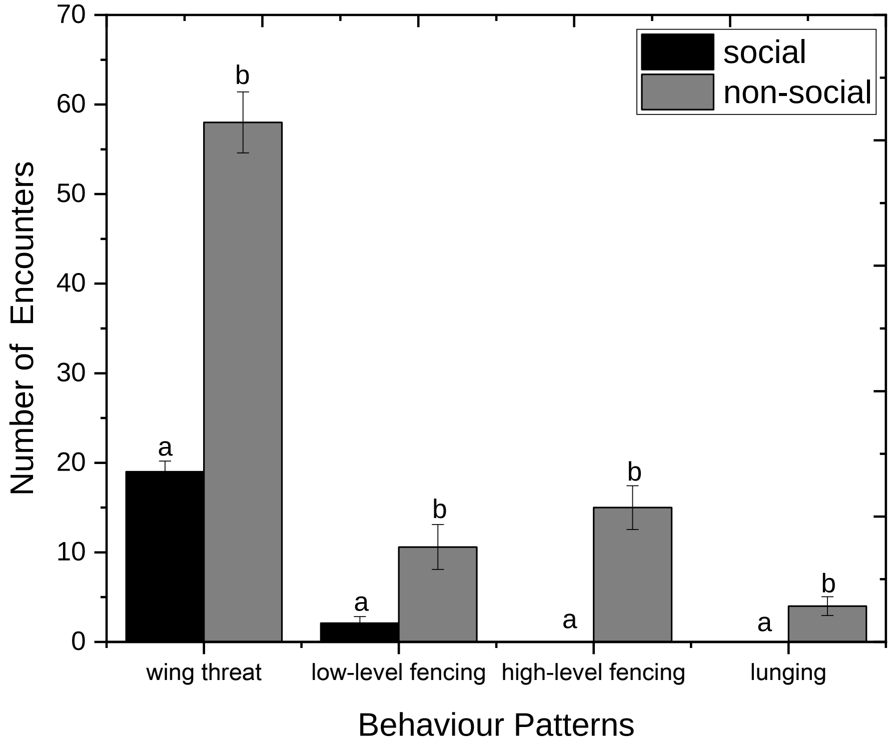

Supplementary Fig. S1: Effect of socialization on aggression of *D. suzukii* non-social and social males. Aggression was quantified with the frequency occurrence of behaviour patterns, which is the number of encounters within 3600 s. Social in black bars and non-social in grey bars (mean ± s.d) (n=25). Statistical differences were evaluated by independent samples T-test with $p<0.05$. Different letters indicates significant differences.

Supplementary Fig. S2

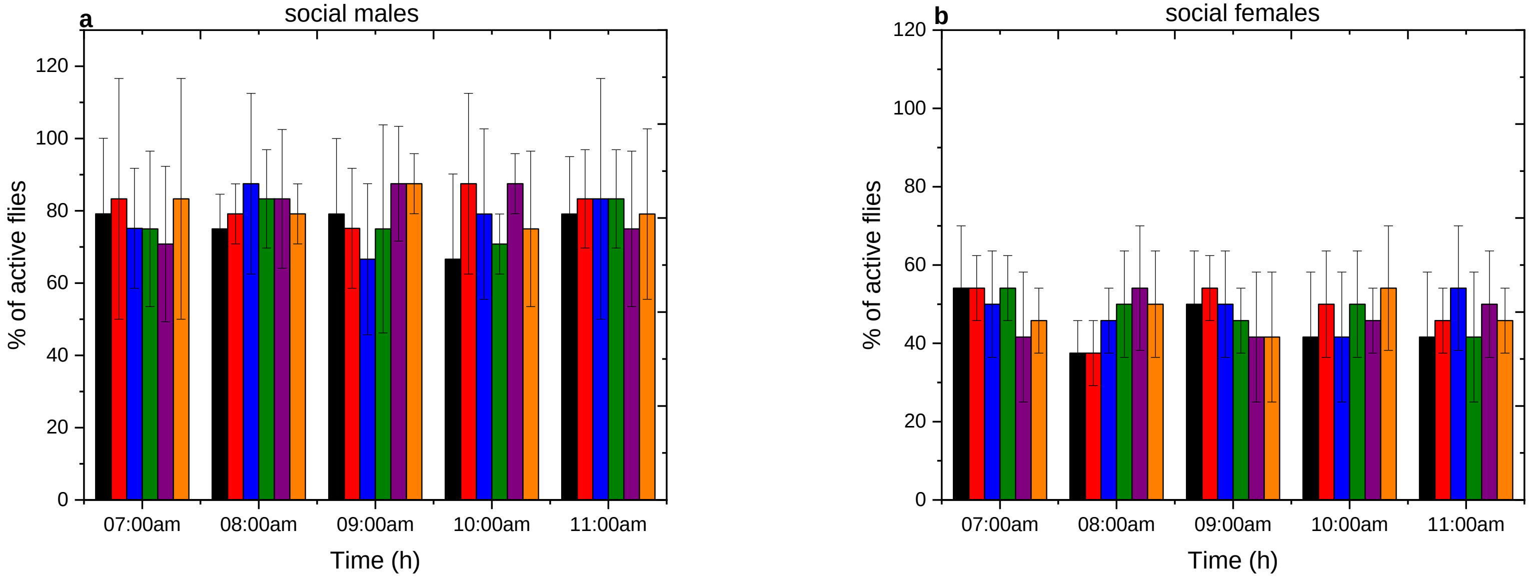

Supplementary Fig. S2: Locomotion activity of *D. suzukii* social males and females. Activity was scored as the % of active flies within the 60 s observation period. (mean ± s.d) (n=24). Locomotion was tested for a) social males and b) social females in 6 different ages (1d-black, 2d-red, 3d-blue, 4d-green, 5d-purpple and 6d-orange) during five hours after beginning of photophase (07:00 am to 11:00 am). There is little statistical difference (One-Way ANOVA social males: $F(29, 90)=0.83$ $p=0.71$; social females: $F(29, 90)=0.84$ $p=0.71$).
